# Sunlight driven photocatalytic degradation of ciprofloxacin over a dual S-scheme based BiOCl/Bi_2_WO_6_/g-C_3_N_4_ ternary heterojunction

**DOI:** 10.1039/d6ra06114a

**Published:** 2026-08-24

**Authors:** Laxmipriya Pradhan, Mano Ranjan Barik, Sushanta Kumar Badamali

**Affiliations:** a Department of Chemistry, Utkal University Vani Vihar Bhubaneswar 751004 Odisha India skbuche@utkaluniversity.ac.in

## Abstract

The contamination of fluoroquinolone antibiotics in wastewater, particularly ciprofloxacin (CIP), poses significant environmental and health hazards, as these are resistant to conventional treatment methods. In the current study, a highly effective dual S-Scheme-based heterojunction photocatalyst, comprising BiOCl, Bi_2_WO_6_, and g-C_3_N_4_, was designed *via* a simple hydrothermal method. The developed material was comprehensively characterized using various physicochemical techniques such as XRD, FTIR, FESEM, HRTEM, XPS, N_2_ sorption analysis, TGA, DR UV-vis, and PL, confirming the successful assembly of a well-integrated nanostructure with an optimised band configuration favourable for visible-light absorption. Under sunlight exposure, the BiOCl/Bi_2_WO_6_/g-C_3_N_4_ heterojunction (BWC) demonstrated excellent photocatalytic activity, achieving 96% degradation of CIP within 90 min, outperforming the individual components. The outstanding activity was ascribed to the cooperative effect of dual S-Scheme charge transfer, which enhanced charge separation and preserved their strong redox abilities. Heterogeneity and reusability studies were performed, and the developed catalyst showed excellent stability up to the fifth run with consistent performance. Kinetic study showed a first-order degradation pattern, while radical trapping tests detected superoxide radicals as the major reactive oxygen species. Based on fragments obtained from LCMS analysis, a plausible mechanism of CIP degradation was established.

## Abstract 2

Herein, we report the development of a cost-effective dual S-scheme BiOCl/Bi_2_WO_6_/g-C_3_N_4_ (BWC) heterostructure photocatalyst for the efficient removal of the ciprofloxacin antibiotic.

## Graphical abstract

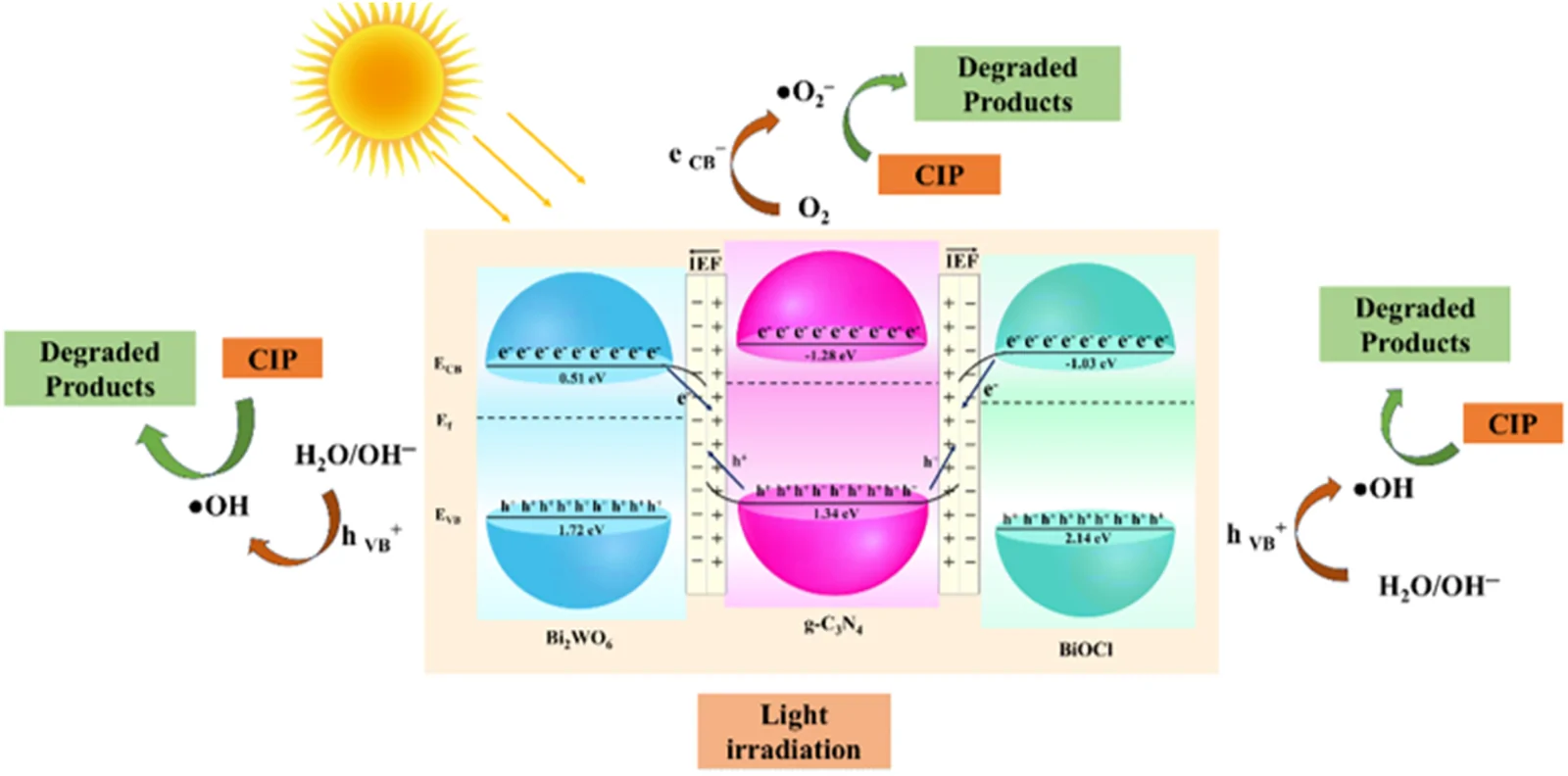

## 1. Introduction

Carbon based compounds have become an integral part of day-to-day life owing to their widespread utilization in pharmaceuticals, agriculture, food processing, and industrial manufacturing. The continuous expansion of chemical industries to meet growing societal demands has significantly increased the liberation of toxic and persistent substances into the atmosphere. The continuous accumulation of these pollutants has turned out to be a major issue due to their toxicity and risks to the environment. Among these, pharmaceuticals, especially antibiotics, have emerged as a class of contaminants due to their extensive usage and persistence.^1,2^ Antibiotics play an indispensable role in modern society in the prevention and treatment of bacterial infections. Overuse of these compounds causes a wide range of harmful effects on living organisms. Pharmaceuticals are found in water at very low concentrations, often ng L^−1^ to µg L^−1^.^3,4^ For example, ciprofloxacin (CIP), a commonly used fluoroquinolone antibiotic, is widely prescribed for treating a variety of bacterial infections, such as respiratory tract infections, digestive system infections, and urinary tract infections.^5–7^ However, the problem is that fluoroquinolones are difficult to metabolize, resulting in 20 to 80% of the drug being excreted into the environment. Based on the official notification of the Ministry of Environment, Forest and Climate Change, India (New Delhi, 23rd January 2020), the permissible limit values of CIP are 0.02 ppm.^8^ Elevated concentrations above this value generate significant risks to the aquatic environment, as these compounds exhibit greater chemical stability and are resistant to natural decay. Previous studies have reported that even at trace concentrations, CIP can promote the proliferation of antibiotic-resistant bacteria and resistance genes.^9,10^ Consequently, the effective remediation of these organic pollutants from aqueous environments is imperative to mitigate ecological degradation and ensure the long-term stability of aquatic ecosystems.

Various treatment methods such as adsorption, coagulation, precipitation, froth flotation, and advanced oxidation processes (AOPs) are proven effective at removing antibiotics from contaminated water; however, these processes are constrained by their high energy consumption, high cost, and discharge of harmful byproducts.^11,12^ Among the AOPs, photocatalysis stands out because it is a green, clean, sustainable, and cost-effective technique.^13,14^ The photocatalytic mechanism involves light absorption by a semiconductor, promoting the formation of electron–hole pairs that interact with oxygen and water to generate reactive organic species (ROS), such as the hydroxyl (˙OH) and the superoxide radical (O_2_˙^−^). Then, these ROS attack organic moieties, leading to their degradation into smaller and less complex intermediates.^15–17^

In recent years, various photocatalysts such as metal oxides, metal sulphides, bismuth-based materials, and two-dimensional materials have been extensively investigated for wastewater purification due to their light-harvesting properties and ability to generate highly reactive radicals.^18^ Most traditional catalysts are active under ultraviolet (UV) radiation, which is less than 5% of the incoming sunlight. The remaining 95% (visible and infrared light) passes through the catalyst without being utilized.^19^ Additionally, they cause the rapid e–h pair recombination, as they recombine much faster in the range of picoseconds to nanoseconds.^20,21^ Conversely, surface redox reactions occur on a timescale of microseconds to milliseconds, indicating that recombination dominates over photocatalytic reactions. Due to recombination, the number of available charge carriers for the photocatalytic reaction is reduced. Also, the amount of energy gained during photon absorption is lost as phonons and photoluminescence emission. This leads to poor charge separation and low quantum efficiency.^22^ To mitigate these shortcomings, strategies such as composite fabrication, metal or non-metal doping, and heterojunction construction have been implemented to minimize the e–h pair recombination, thereby improving the overall activity.^23^ Among these approaches, heterojunction-based photocatalysts are important as it significantly enhances charge separation.^24^ When two semiconductors with different band structures are brought into intimate contact, at the junction of two materials, an internal electric field (IEF) is created. This IEF drives the recombination of less reactive electrons and holes while preserving the charge carriers with strong redox potentials. Consequently, heterojunctions extend the lifetime of reactive electrons, and holes also increase their availability for surface reactions, leading to enhanced catalytic performance.^25^ Additionally, heterojunctions can broaden light absorption by combining materials with different band gaps and can provide more favourable redox potentials for specific reactions. Heterojunctions can be classified based on the relative alignment of the energy bands of the coupled materials and the way charge carriers migrate across the interface. The S-scheme (Step-scheme) heterojunction is a highly efficient photocatalytic system consisting of an oxidation photocatalyst (OP) and a reduction photocatalyst (RP) with a staggered energy band structure. When these materials contact, a built-in electric field and interfacial band bending are established, which act as a driving force to selectively recombine weak charge carriers at the interface. This step-like transfer pathway ensures that only the highly reactive electrons and holes are preserved on the surfaces, thereby enhancing the overall redox ability of the catalyst for reactions like hydrogen production or pollutant degradation.^26^ A dual S-scheme heterojunction is a ternary photocatalytic system comprising three semiconductors that form two distinct S-scheme interfaces to create a multi-channel charge transfer network. By strategically aligning an oxidation photocatalyst, a reduction photocatalyst, and a third component, the system utilizes two internal electric fields to simultaneously eliminate weak charges at both interfaces. This configuration effectively doubles the charge separation efficacy while preserving the maximum redox potential of the strong electrons and holes for complex reactions.

Some of the common photocatalysts studied are metal oxides, such as for example TiO_2_, ZnO, WO_3_, Fe_2_O_3_, metal sulphides, bismuth-based photocatalysts such as Bi_2_WO_6_, BiVO_4_, and BiOX (where X = F, Cl, Br, I), and 2D polymeric photocatalysts such as g-C_3_N_4_ and rGO available in open literature.^27^ There are many synthetic techniques available for these materials, such as hydrothermal, sol–gel, and precipitation methods.^28^ Among the available techniques, hydrothermal synthesis offers several distinct advantages over other conventional synthesis methods. The hydrothermal process emphasises reactions mostly carried out in the presence of water and autogenous pressure by raising the temperature in a sealed autoclave to grow crystals from aqueous solutions with high crystallinity and high phase purity, enabling the creation of high-surface-area nanostructures like nanosheets or nanotubes that maximize active sites. Furthermore, it facilitates *in situ* heterojunction growth, creating the intimate atomic contact necessary for efficient charge transfer in S-scheme systems.^29^ Among the studied photocatalysts, bismuth oxychloride is a layered Aurivillius-type semiconductor whose unique [Bi_2_O_2_]^2+^ slabs separated by Cl^−^ layers generate a strong internal electric field, effectively suppressing electron–hole recombination. Bismuth tungstate is a semiconductor photocatalyst with a narrow bandgap that absorbs in the visible region.^30^ The material is nontoxic, chemically stable, and environmentally friendly. Graphitic carbon nitride is a metal-free polymeric semiconductor photocatalyst with a moderate bandgap (∼2.7 eV), facilitating absorption of visible radiation. Its layered π-conjugated structure facilitates charge transport and chemical stability.^31^ The combination of all these semiconductors significantly enhances the light-harvesting property and promotes effective charge transfer, leading to efficient mineralization of CIP.

A comprehensive review of the literature reveals that a broad spectrum of photocatalytic materials and experimental conditions have been explored for the degradation of ciprofloxacin, as summarized in Table TS1 (SI). Wang *et al.* have taken g-C_3_N_4_/TiO_2_ powder as the catalyst with a 150 W tungsten lamp, and received 95% efficiency. However, the problem lies in the higher catalyst dosage (1 g L^−1^) and the non-renewable irradiation source. Mchiri *et al.* have studied photocatalytic degradation by using g-C_3_N_4_/Bi_2_WO_6_ and solar radiation, showing a 91% of efficiency within a period of 6 h. Similarly, B. Yao *et al.* used Zn/Cu_2_O as the catalyst and a 500 W halide lamp, which was irradiated for 4 h to degrade 94.6% of CIP. However, the source used and the exposure duration as of concern with respect to sustainability. Collectively, these studies highlight persistent limitations in non-renewable energy sources, higher reaction time, and high dosage of catalyst. Hence, there is a high demand for the enrichment of a sustainable photocatalytic system that can show a superior rate of degradation using minimal dosage and reduced treatment time.

The objective of this study is to develop a bismuth-based highly efficient ternary dual S-scheme-based heterostructured photocatalyst for the efficient mineralization of CIP under minimal reaction conditions. The designed photocatalyst aims to enhance visible-light utilization, facilitate effective interfacial charge separation, and promote directional charge transfer, thereby minimizing the recombination of e–h pairs. In addition, the study seeks to gauge the photocatalytic activity under environmentally relevant conditions, including low catalyst dosage, reduced reaction time, and natural sunlight irradiation, to ensure sustainability and practical applicability. The stability, reusability, and resistance to photocorrosion of the catalyst are also systematically investigated to confirm its suitability for long-term operation. We also aim to analyse the obtained fragments during the photocatalytic reaction using the LC-MS technique and then provide a plausible degradation pathway for CIP mineralization. Overall, this work aims to provide an eco-friendly and economically viable approach for the development of a robust photocatalyst capable of utilizing solar energy for efficient wastewater treatment.

## 2. Experimental

### 2.1 Reagent and catalyst

All precursors employed in this study were of analytical-grade purity and used as received, without further purification. Floxip 500 (ciprofloxacin tablets IP, 500 mg), supplied by Abbott India Limited, was employed as the model pollutant in this investigation. Urea (99.5%, Sisco Research Laboratory, India), hydrochloric acid (35%, Merck), bismuth nitrate pentahydrate (98.5%, Sisco Research Laboratory, India), sodium tungstate pentahydrate (99%, Sisco Research Laboratory, India), sodium chloride (99.9%, Sisco Research Laboratory, India), ethylene glycol (98%, Sisco Research Laboratory, India), and glacial acetic acid (100%, Sigma Aldrich) were used for the preparation of the catalyst. All the reactions were carried out in aqueous media, using double distilled water.

### 2.2 Synthesis of g-C_3_N_4_

Graphitic carbon nitride (g-C_3_N_4_) was derived through the thermal polymerization of urea.^32^ In a typical synthesis, 200 mmol of urea was placed in a 50 mL silica crucible and sealed. It was calcined at 550 °C, by raising the temperature from room temperature at a heating rate of 5 °C min^−1^ under an air atmosphere and hold for 3 h. The obtained yellow powder was washed using deionized water and 1 M HCl for the elimination of the remaining alkaline species. After filtration, the residue was dried at 70 °C for 8 h. The ammonium salts, which may have formed in the dried powder during acid treatment, were removed by heating the powder for 3 h at 400 °C to obtain the sheet-like g-C_3_N_4_.

### 2.3 Synthesis of Bi_2_WO_6_

**Scheme 1:**
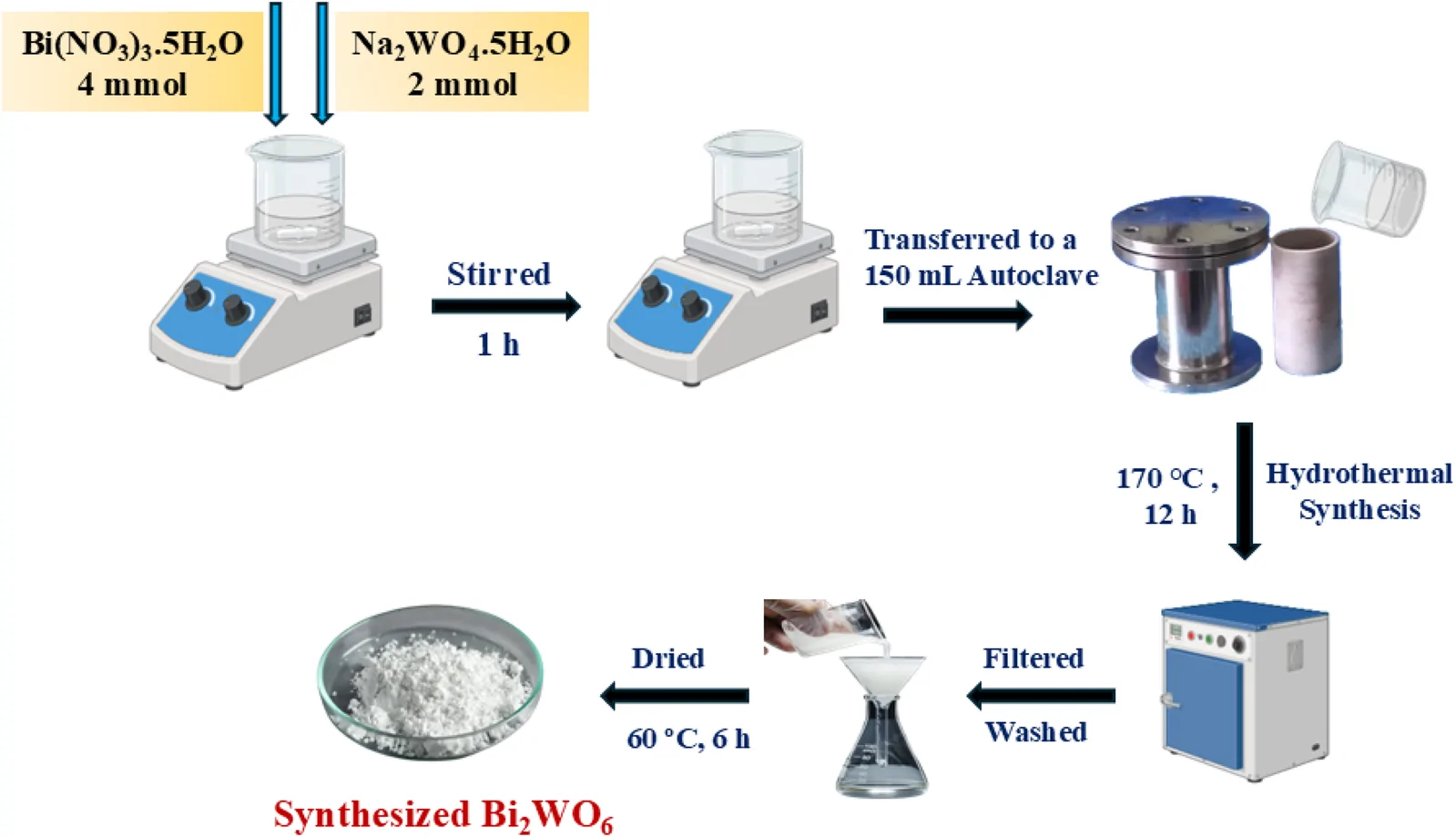
Synthesis of Bi_2_WO_6_.

Bi_2_WO_6_ was synthesized through a hydrothermal route as presented in Scheme 1. Initially, 4 mmol of Bi(NO_3_)_3_·5H_2_O and 2 mmol of Na_2_WO_4_·5H_2_O were dissolved in deionized water and stirred for 1 h to ensure complete mixing and homogeneity of the precursor solution. The resulting suspension was taken in a 100 mL autoclave and maintained at 170 °C for 12 h to facilitate the formation of Bi_2_WO_6_ crystals. After cooling, the obtained precipitate was filtered and repeatedly washed with deionized water to remove residual ions and impurities. Finally, the product was dried at 60 °C for 6 h, yielding a white coloured Bi_2_WO_6_ powder.

### 2.4 Preparation of heterojunction (Bi_2_WO_6_/g-C_3_N_4_/BiOCl)

**Scheme 2:**
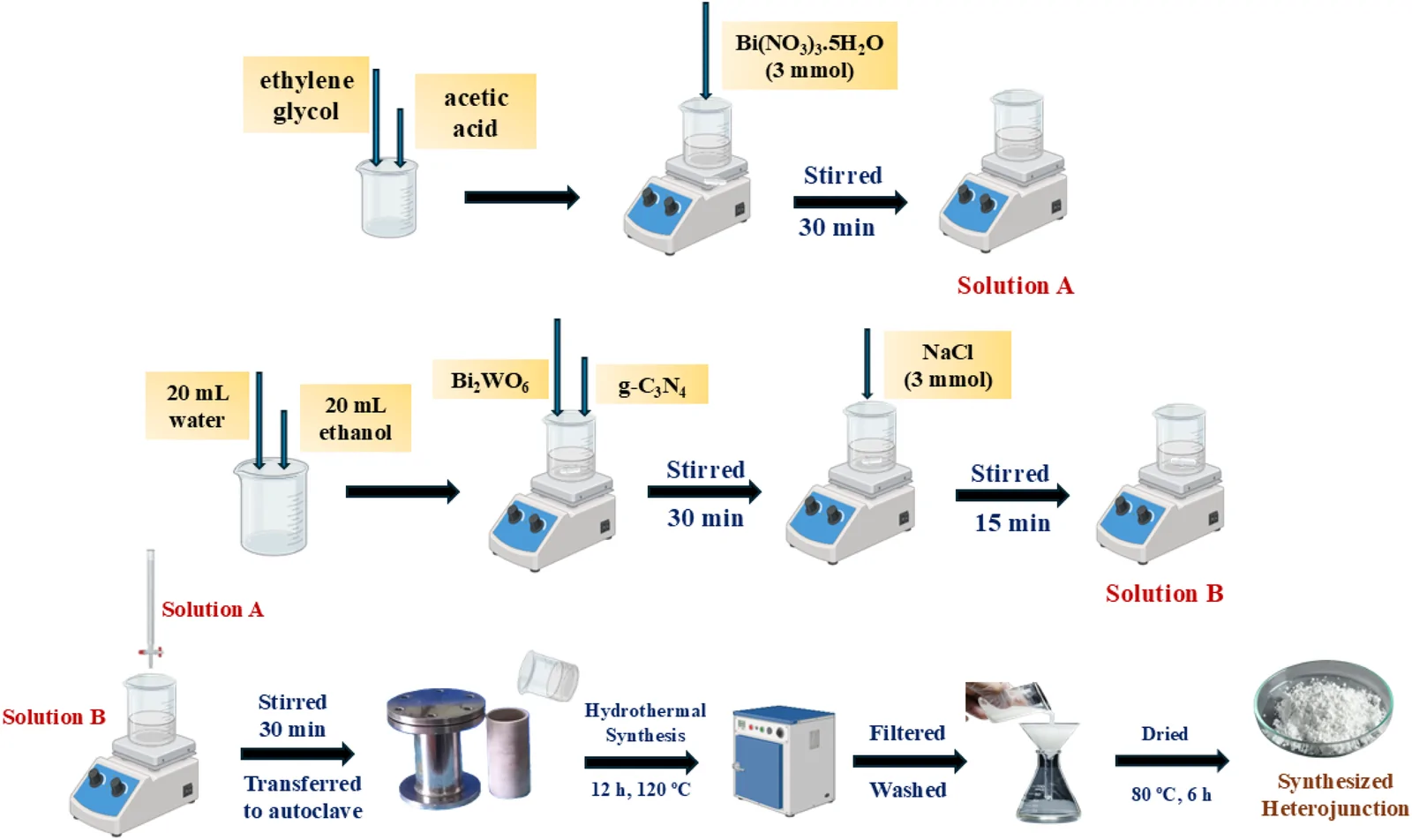
Preparation of BWC heterojunction.

The synthesis of the BWC heterojunction is presented in Scheme 2. About 3 mmol of Bi(NO_3_)_3_·5H_2_O was placed in a 40 mL solution of ethylene glycol and acetic acid with 1 : 1 v/v ratio, under constant stirring, and it was named as solution A. In another beaker, a desired predetermined amount of Bi_2_WO_6_ and g-C_3_N_4_ were taken in 40 mL of water–ethanol mixture (1 : 1 v/v) solution and stirred for 20 min to prepare a 3 : 2 : 1 w/w molar composition of BiOCl : Bi_2_WO_6_ : g-C_3_N_4_. Then, 3 mmol of NaCl was added to it and stirred for 20 min to obtain solution B, followed by the gradual addition of solution A to B. After an additional 30 min of stirring, the homogeneous suspension was transferred to a 100 mL autoclave and kept at 120 °C for 12 h to facilitate crystal growth. The crystallized solid samples were obtained by centrifugation, followed by repeated washing and drying at 80 °C for 6 h to get the final BWC heterojunction. Similarly, pure BiOCl was also synthesized in the same process without adding Bi_2_WO_6_ and g-C_3_N_4_.

### 2.5 Characterization

The synthesized catalysts were comprehensively characterized to analyze the structural integrity, morphological, elemental composition, electronic, and optical properties. The crystal structure of BWC was examined using a PANalytical X-ray diffractometer equipped with Cu Kα radiation (*λ* = 1.5406 Å). The chemical structure and surface functionalities of the prepared materials were characterized using Fourier transform infrared spectroscopy (Shimadzu, Japan). Field Emission Scanning Electron Microscope (Zeiss, Germany, 5 kV) for the analysis of surface morphology and particle distributions. The nanoscopic morphology and lattice fringes were analysed by using a High-Resolution Transmission Electron Microscope (PHILIPS CM 200) at 5 kV. The electronic states and surface chemical composition of the BWC heterostructure were examined using an Omicron ESCA X-ray photoelectron spectroscopy system (Oxford Instruments, Germany). Nitrogen adsorption–desorption measurements were conducted using a Quantachrome analyzer to assess the surface area, pore volume, and pore size distribution of BWC. Before analysis, the sample underwent degassing at 180 °C for 8 h. TG-DTA (NETZSCH STA 449 F5, Germany) was used to gauge the thermal stability of the catalyst. UV-vis DR spectroscopy (PerkinElmer, Germany) scanning between 200 and 800 nm was used to examine the light-absorption properties and estimate the bandgap values of the material. The emission properties of the BWC heterostructure were evaluated by photoluminescence spectroscopy at room temperature using an Edinburgh FLS 980S system operated with an excitation wavelength of 320 nm. Total organic carbon was measured in a TOC-L Shimadzu analyzer after the complete oxidation of the CIP molecules. The analysis of the CIP fragments and reaction intermediates obtained during the photocatalytic reaction was evaluated using an Agilent 6530 Q-TOF LC MS system.

### 2.6 Catalytic activity studies

Photocatalytic studies were carried out under sunlight on bright and clear days between 11 AM to 2 PM to ensure constant and intense irradiation. The reactions were exposed to natural sunlight (Bhubaneswar, Odisha, India; latitude: 20.3007, longitude: 85.8425), with an average solar irradiance ranging from 5.0 to 5.1 kWh m^−2^ day^−1^, and an incident light intensity of ∼94–101 mW cm^−2^. In typical degradation run, 20 mg of catalyst was introduced into 50 mL of a 20 mg L^−1^ CIP aqueous solution. To ensure equilibrium adsorption of CIP on the photocatalyst surface, the above reaction mixture was stirred in the dark for 20 min before irradiation. After achieving equilibrium, the reaction mixture was subjected to solar radiation for 90 min to establish the degradation reaction. After each 15 min intervals, 5 mL of the reaction suspension was withdrawn and centrifuged for 12 min to remove the catalyst from the solution. The residual CIP was measured by monitoring the fall in absorbance values during the reaction at its characteristic wavelength (*λ*_max_ = 274 nm) using a UV-vis spectrophotometer (Agilent Cary 100, USA). To establish the optimum operating conditions, the effects of photocatalyst loading and initial CIP concentration were systematically investigated.

The photocatalytic degradation efficiency of CIP was evaluated by using the expression:1
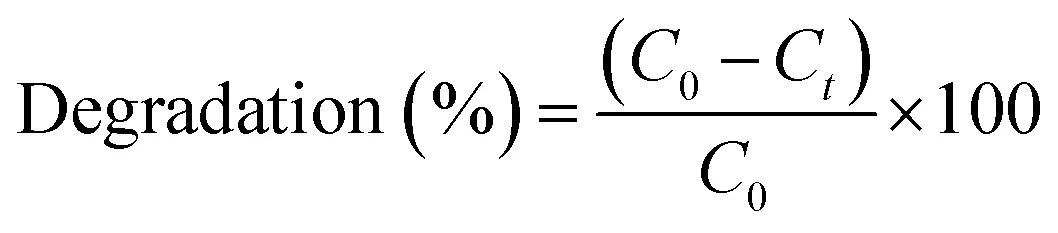
where *C*_0_ and *C*_*t*_ are the concentrations of CIP at the initial and after a definite interval of time *t*, respectively. Since absorbance is directly proportional to concentration, absorbance values were used to calculate degradation efficiency.

The heterogeneity tests were conducted by taking the filtrate of the BWC heterostructure for the degradation study. Initially, the BWC was placed in distilled water and stirred for 90 min under sunlight, and then it was filtered to separate the solid residue from the filtrate. Both parts were individually tested for the degradation study, maintaining the identical reaction conditions. The stability of the catalyst was monitored *via* reusability experiments. After each run, the BWC was recovered by centrifugation and then washed with deionized water to eliminate any surface-adsorbed impurities. The recovered material was then dried and thermally regenerated at 400 °C for 6 h before being reused in subsequent photocatalytic cycles. This procedure was repeated over five cycles to investigate the reusability and retention of photocatalytic activity of the catalyst.

## 3. Results and discussion

### 3.1 Catalyst and characterization

#### 3.1.1 XRD analysis

**Fig. 1:**
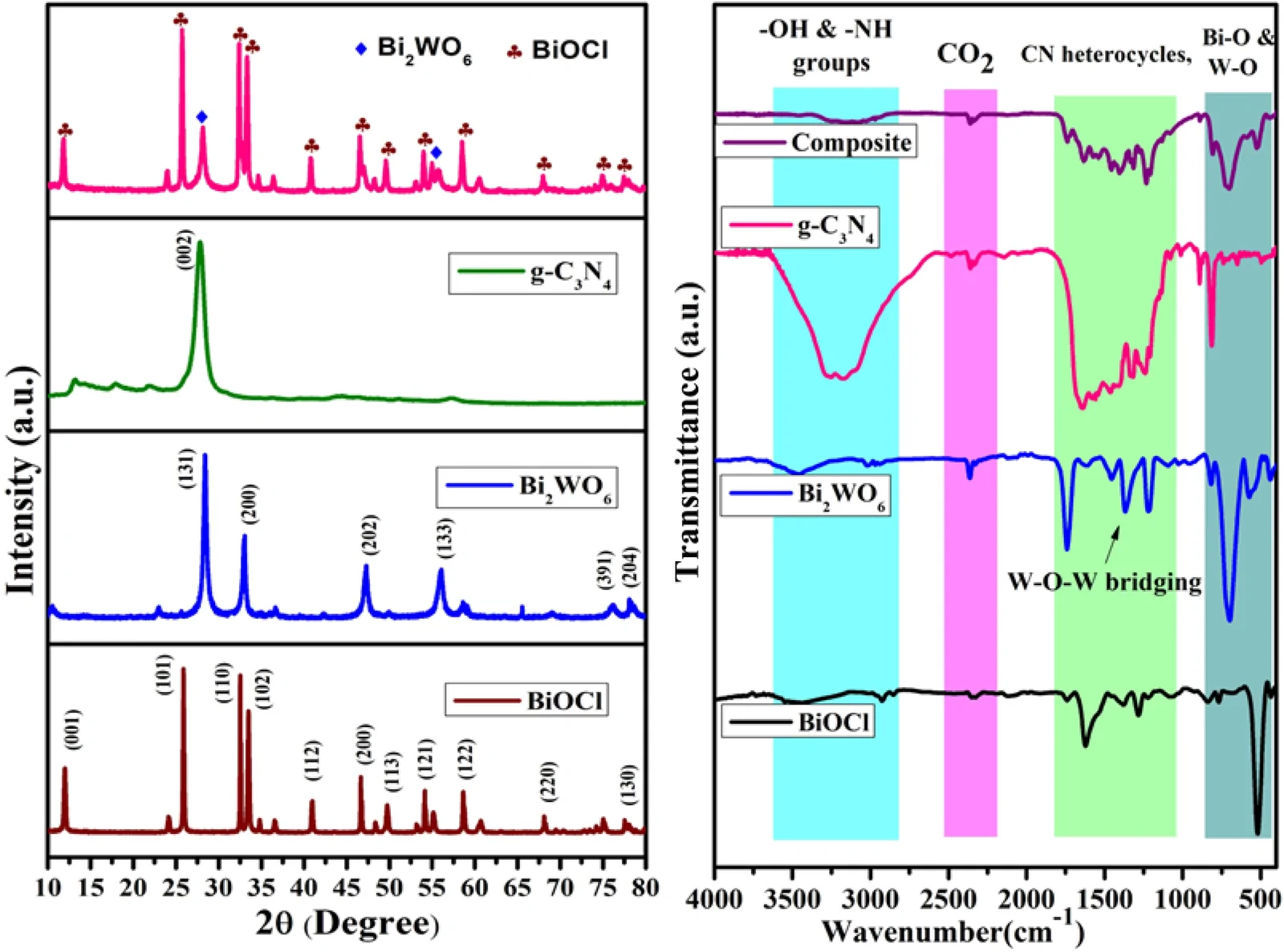
XRD patterns and FTIR spectra of g-C_3_N_4_, Bi_2_WO_6_, BiOCl, and the BWC heterostructure.

The detailed XRD patterns obtained are depicted in Fig. 1a. As it can be observed, BiOCl shows a well-defined characteristic reflections of the tetragonal phase, with pronounced reflections at 2*θ* values of 12.1, 25.8, 32.5, 33.5, 40.9, 46.5, 49.6, 54.1, 58.6°, corresponding to the (001), (101), (110), (102), (112), (200), (113), (121), and (122) crystal planes, respectively confirming its high degree of crystallinity (JCPDS: 73-2060).^33,34^ Bi_2_WO_6_ demonstrates distinct and intense diffraction peaks corresponding to the orthorhombic phase, along with the (131), (200), (202), (133), (391), (204) crystal planes at 2*θ* values of 28.3, 33.1, 47.1, 55.9, 76.2, 78.1°, respectively (JCPDS: 39-0256).^35^ The g-C_3_N_4_ sample displays a characteristic diffraction peak (002) at a 2*θ* value of 27.4°, indicating the interlayer stacking within the graphitic network.^32^ The BWC heterostructure shows the characteristic peaks of all the individual components, BiOCl, Bi_2_WO_6_, and g-C_3_N_4_, indicating successful fabrication of the components.

#### 3.1.2 FTIR analysis

The FTIR spectra of the BWC heterostructure exhibited the characteristic vibrational signatures of BiOCl, Bi_2_WO_6_, and g-C_3_N_4_, indicating the successful formation of the ternary composite (Fig. 1b). The absorption band centered at 520 cm^−1^ corresponds to the Bi–O stretching vibration, whereas the peak at 706 cm^−1^ is assigned to the W–O stretching mode, confirming the presence of the BiOCl and Bi_2_WO_6_ phases, respectively.^36,37^ The prominent bands appearing within the 1200–1650 cm^−1^ region are due to the stretching vibrations of C–N and C=N bonds in the heptazine framework of g-C_3_N_4_. A weak feature observed near 2340 cm^−1^ is assigned to adsorbed atmospheric CO_2_. Moreover, the broad absorption envelope extending from 3000 to 3600 cm^−1^ is related to O–H and/or N–H stretching vibrations, confirming the presence of surface hydroxyl groups, adsorbed water molecules, and residual amino functionalities on the material surface.^38^

#### 3.1.3 FE SEM analysis

**Fig. 2:**
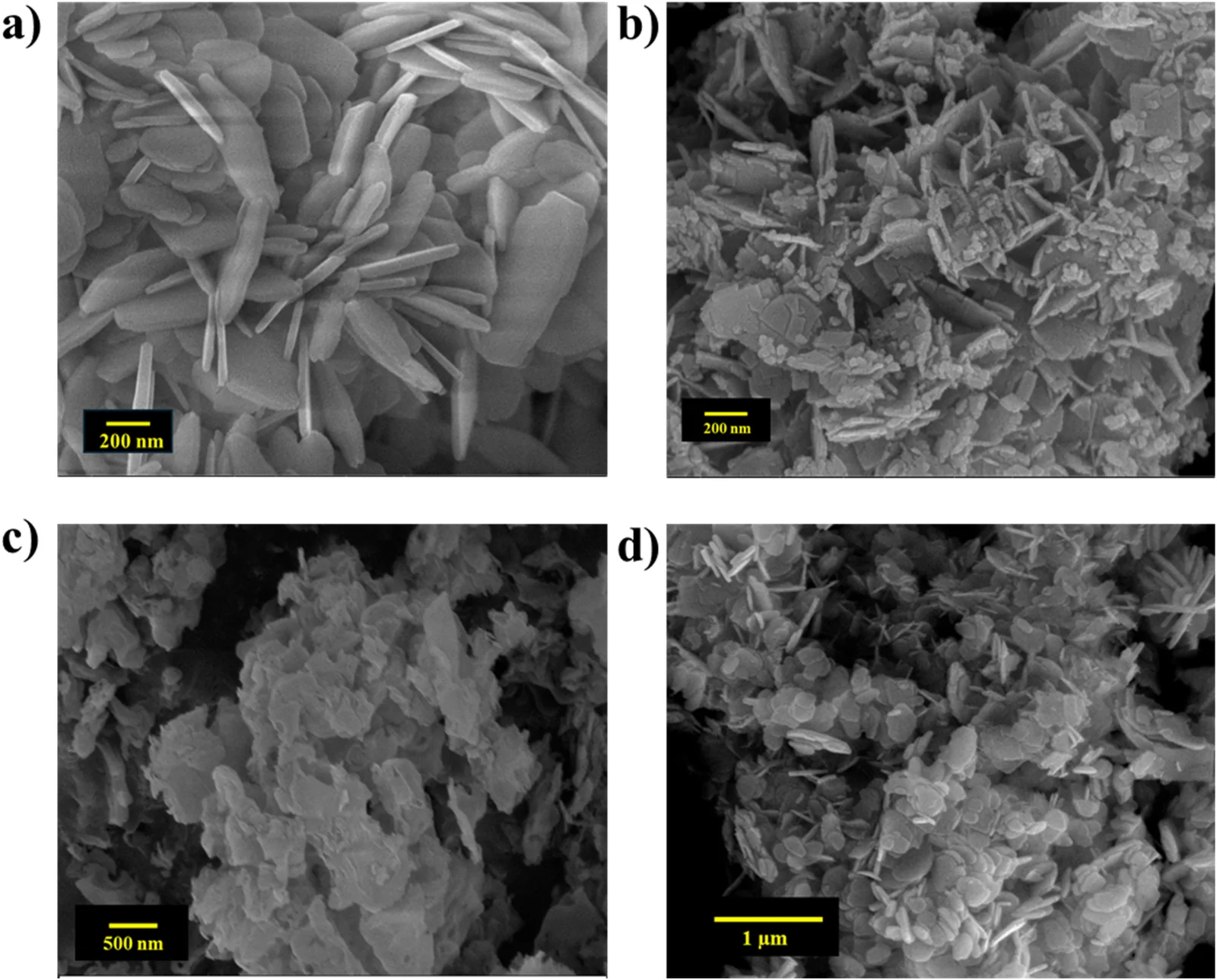
FE SEM micrographs of BiOCl (a), Bi_2_WO_6_ (b), g-C_3_N_4_ (c), and BWC (d).

The surface morphological features of the developed heterostructures were characterized, and the micrographs are shown in Fig. 2. Pure BiOCl displays flake-like morphology with a flat and smooth surface (Fig. 2a). These are oriented randomly with well-defined layered structures.^39^ In the case of Bi_2_WO_6_ (Fig. 2b), nanosheets decorated by granular nanoparticles were observed.^40^ The aggregation of nanosheets with attached nanoparticles forms a rough and porous architecture, which contributes to a greater surface area and provides adequate active sites, facilitating improved photocatalytic performance. Whereas g-C_3_N_4_ (Fig. 2c) exhibits a layered, sheet-like configuration due to its graphitic heptazine units. Such morphology is commonly observed in two-dimensional materials or layered compounds, where individual sheets tend to restack due to van der Waals interactions.^38^ These structures indicate that the material offers a large surface area to hold the substrate molecules. The ternary BWC heterostructure (Fig. 2d) exhibits a well-integrated construction in which BiOCl and Bi_2_WO_6_ are uniformly decorated across the g-C_3_N_4_ surface, ensuring the heterojunction interface formation among the individual components. The EDX pattern and elemental mapping reveal the elemental presence of Bi, W, Cl, O, C, and N in the composite, confirming the effective construction of the BWC heterostructure (Fig. S1, SI).

#### 3.1.4 HR TEM analysis

**Fig. 3:**
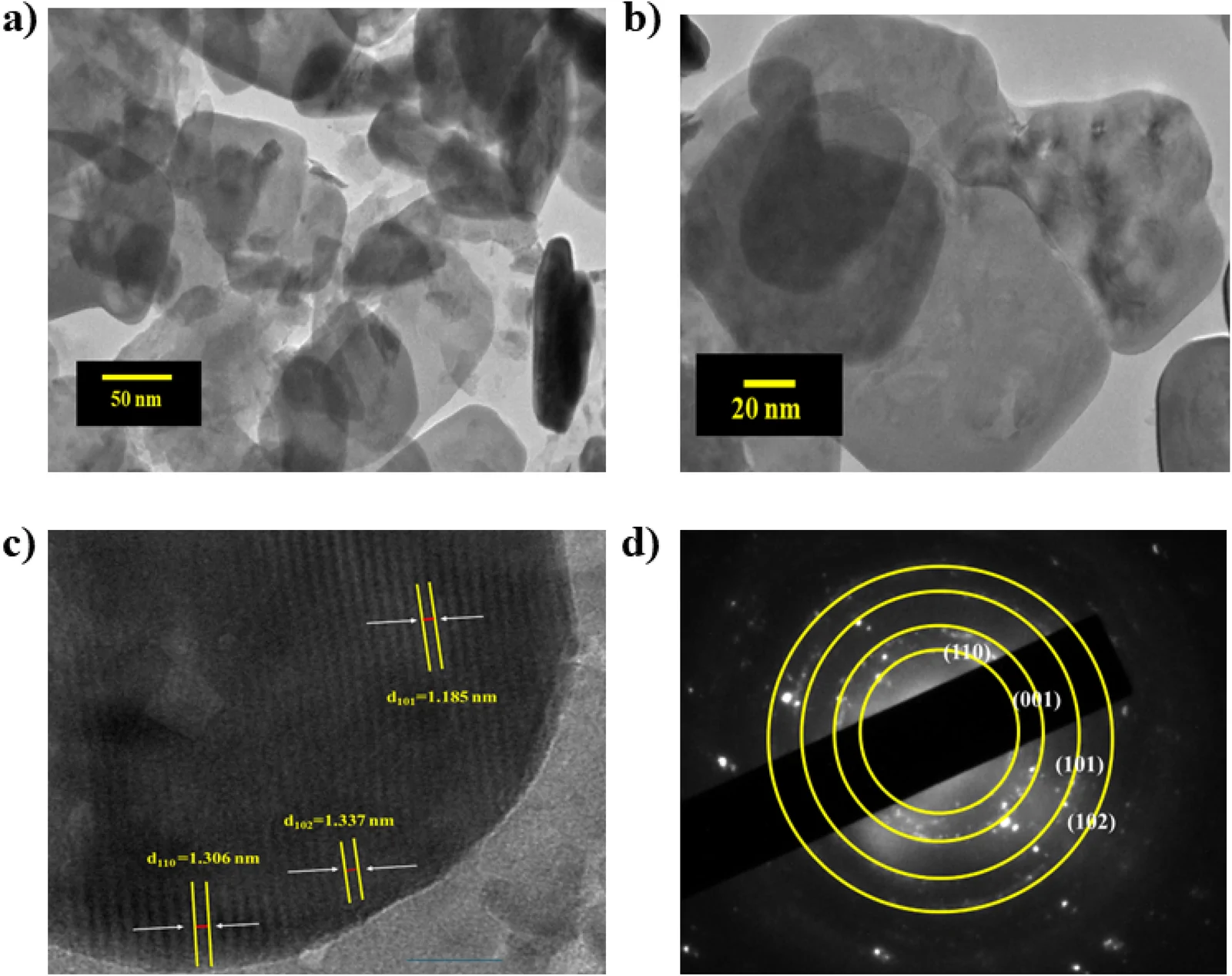
HR-TEM images of BWC at different resolutions (a) and (b), lattice fringe analysis (c), and the corresponding SAED pattern (d).

The high-resolution TEM images depicted in Fig. 3 furnish direct evidence of the structural arrangement and interfacial contact among the components of the BWC heterostructure. Fig. 3a and b depict the heterojunction consisting of nanoparticles embedded in a thin layered framework, which confirms the effective assembly of BiOCl, Bi_2_WO_6_, and g-C_3_N_4_ within the synthesized heterostructure. Higher-magnification images in Fig. 3c display clear lattice fringes with well-defined interplanar distances. The estimated *d*-spacings of 1.33, 1.30, and 1.18 nm correspond to (102), (110), and (101) planes, respectively, thereby confirming the polycrystalline structure of the material. The SAED pattern in Fig. 3d also shows similar places, which is also in accordance with the XRD patterns.

#### 3.1.5 XPS analysis

**Fig. 4:**
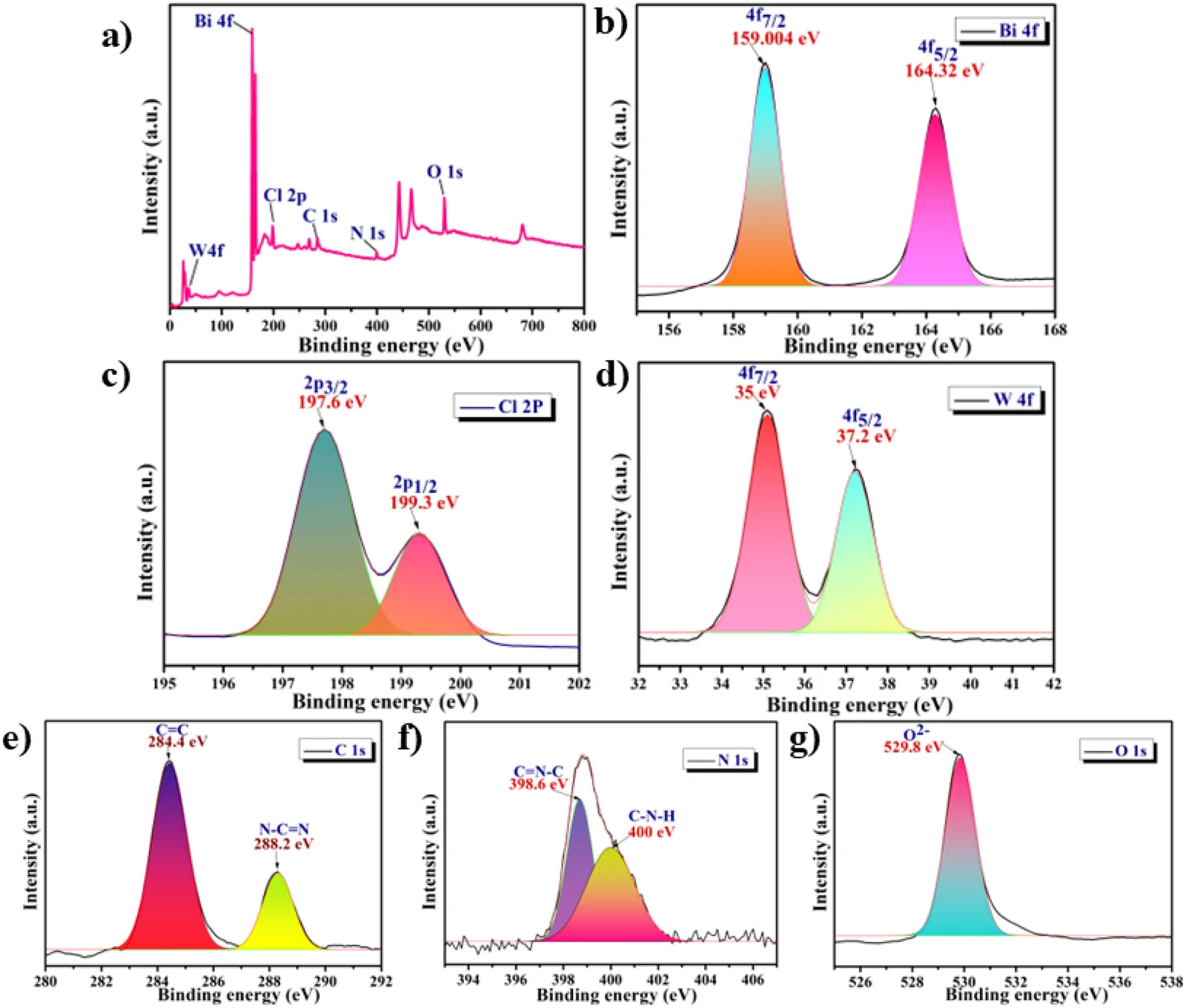
XPS analysis of the BWC heterostructure showing the survey spectrum (a) and the corresponding high-resolution spectra of Bi 4f (b), Cl 2p (c), W 4f (d), C 1s (e), N 1s (f), and O 1s (g).

The surface elemental composition and chemical states of the BWC heterostructure were analysed by X-ray photoelectron spectroscopy (XPS). The survey spectrum (Fig. 4a) reveals the presence of Bi, W, Cl, C, N, and O elements, confirming the coexistence of BiOCl, Bi_2_WO_6_, and g-C_3_N_4_ within the heterostructure. No additional impurity-related peaks were detected, demonstrating the high purity of the synthesized material. The high-resolution Bi 4f spectrum (Fig. 4b) exhibits two characteristic peaks located at 159.0 and 164.3 eV, corresponding to Bi 4f_7/2_ and Bi 4f_5/2_, respectively. The observed spin–orbit splitting is characteristic of Bi^3+^ species, confirming the trivalent oxidation state of bismuth in the heterostructure.^41^ The Cl 2p spectrum displays two peaks at 197.6 and 199.3 eV, attributed to Cl 2p_3/2_ and Cl 2p_1/2_, respectively (Fig. 4c). These features are characteristic of lattice chloride ions in the BiOCl structure, demonstrating the preservation of the BiOCl phase after heterojunction formation.^42^ As shown in Fig. 4d, the W 4f spectrum can be deconvoluted into two peaks centered at 35.0 and 37.2 eV, assigned to W 4f_7/2_ and W 4f_5/2_, respectively. These binding energies are typical of W^6+^ species and verify the presence of tungsten in the Bi_2_WO_6_ lattice.^43^ The C 1s spectrum shows a dominant peak at 284.6 eV, which is assigned to adventitious carbon (C–C) species (Fig. 4e). Another peak centered at 288.2 eV corresponds to the sp^2^-bonded carbon (N–C=N) environment within the heptazine units of g-C_3_N_4_, confirming the existence of the graphitic carbon nitride framework (Fig. 4f).^32^ In the N 1s spectrum, the peak at 398.6 eV is attributed to the sp^2^-hybridized nitrogen atoms bonded to carbon (C–N=C), whereas the peak at 400.0 eV originates from amino-functional nitrogen species (C–N–H). The O 1s spectrum (Fig. 4g) exhibits a prominent peak centered at 529.8 eV, which is assigned to lattice oxygen (O^2−^) associated with the Bi–O and W–O bonds in BiOCl and Bi_2_WO_6_. The presence of this peak confirms the integrity of the metal–oxygen framework within the heterostructure. These characteristic nitrogen environments further verify the formation of the g-C_3_N_4_ network. These chemical state characteristics together confirm the effective construction of the BWC heterostructure and explain its superior activity by demonstrating the improved separation of the photogenerated charge carriers and increasing the formation of reactive oxidative species.

#### 3.1.6 N_2_ adsorption–desorption studies

**Fig. 5:**
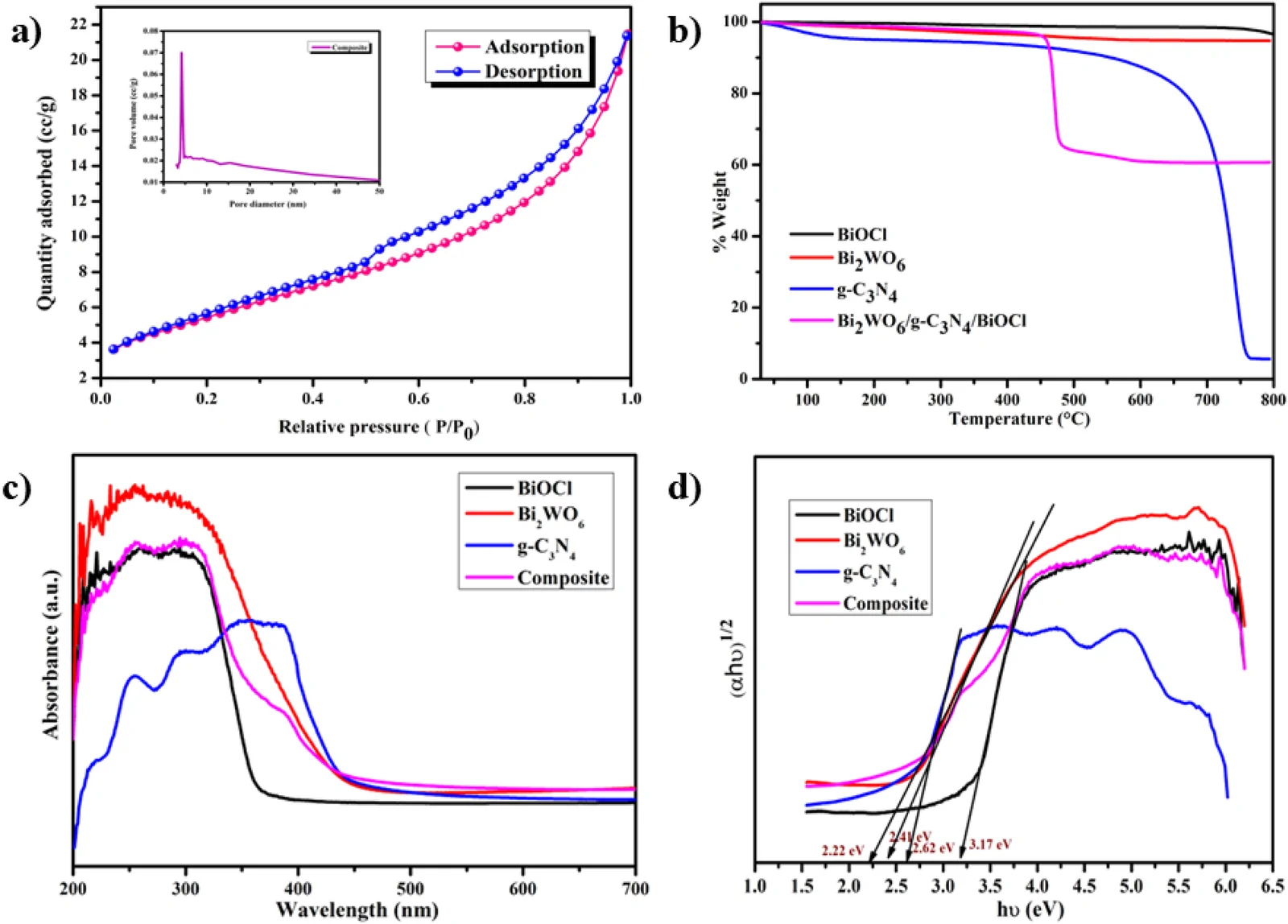
N_2_ adsorption–desorption isotherm (a), TGA analysis of catalysts (b), UV-vis DRS spectra (c), Tauc plot (d).

The surface area and porosity of materials were obtained from N_2_ adsorption–desorption analysis. The BET surface area analysis indicates that the composite has a specific surface area of ∼20 m^2^ g^−1^. From Fig. 5a, a characteristic type IV adsorption isotherm coupled with a broad hysteresis loop was observed for BWC, suggesting the existence of an interconnected meso/macro-porous network. The steady adsorption of nitrogen in the low-pressure region (*P*/*P*_0_ < 0.4) reflects a well-established surface morphology. A pronounced rise in adsorption at higher relative pressures (*P*/*P*_0_ ≥ 0.5 to 0.9), accompanied by a hysteresis loop, is due to capillary condensation within the open framework. Furthermore, the inset in Fig. 5a displays the pore-size distribution curve, which reveals that the material predominantly consists of meso/macro-pores within the structure. BWC exhibited an average pore diameter of ∼6.7 nm, while the corresponding total pore volume was determined to be 0.03 cm^3^ g^−1^. Such textural characteristics facilitate enhanced adsorption of adsorbate molecules and improved diffusion of reactants and intermediates.

#### 3.1.7 TGA analysis

The thermogravimetric analysis (TGA) of BiOCl, Bi_2_WO_6_, g-C_3_N_4_, and BWC is given in Fig. 5b. It offers valuable insight into their thermal stability and decomposition behaviour. For BiOCl, the TGA plot typically shows excellent thermal stability. There is negligible weight loss across the entire temperature range, indicating that bismuth oxychloride remains structurally stable up to 800 °C. Similarly, Bi_2_WO_6_ exhibits high stability. It exhibits ∼5% weight loss, which is ascribed to the removal of surface-bound water molecules. g-C_3_N_4_ shows significant weight loss between 500 and 750 °C, indicating the decomposition of the carbon nitride matrix. At lower temperatures, the composite undergoes a negligible weight loss, which is ascribed to the removal of surface-bound water molecules, followed by a substantial weight loss between 450 and 500 °C, which is associated with the thermal decomposition of the g-C_3_N_4_ component within the composite. The residual mass is due to a thermally robust inorganic framework of BiOCl and Bi_2_WO_6_.

#### 3.1.8 DR UV-vis and photoluminescence studies

The optical properties of the developed materials were evaluated by UV-vis diffuse reflectance spectroscopy (DRS), while their bandgap energies (*E*_g_) were estimated from the corresponding Tauc plots. The obtained results, shown in Fig. 5c and d, reveal the optical absorption characteristics and electronic transition energies of the developed photocatalysts. Pure BiOCl shows a broad absorption edge around ∼360 nm in the UV region, which indicates the wide band gap nature of BiOCl. In the case of Bi_2_WO_6_, an improved visible light absorption edge was obtained in the region ∼440–450 nm. g-C_3_N_4_ displays a pronounced visible light absorption with a characteristic absorption edge near ∼450 nm, due to π → π* electronic excitations within its conjugated framework. The ternary composite demonstrates significantly enhanced and broadened light absorption compared to the individual components. The *E*_g_ of the catalysts was evaluated using the Tauc plots. BiOCl absorbs light in the UV-vis edge because of a wide *E*_g_ of 3.17 eV. Bi_2_WO_6_ and g-C_3_N_4_ show *E*_g_ of ∼2.22 and 2.62 eV, respectively, indicating strong absorption in the visible region. The composite shows an intermediate *E*_g_ of ∼2.41 eV, and the reduction in *E*_g_ value for the composite compared with pure BiOCl suggests improved visible-light absorption due to the interaction between BiOCl, Bi_2_WO_6_, & g-C_3_N_4_. The heterojunction formation among these materials modifies the electronic structure and facilitates better utilization of solar light.

The photoluminescence study of BiOCl, Bi_2_WO_6_, g-C_3_N_4,_ and the BWC is presented in Fig. S2 (SI), which provides vital information about their charge transfer dynamics and recombination characteristics. The individual catalysts demonstrate strong emission peaks corresponding to the fast recombination of e–h pairs. But the BWC heterostructure exhibits a substantial decrease in PL intensity compared to g-C_3_N_4,_ confirming the efficient spatial charge carrier separation between the components and the inhibition of rapid e–h pair recombination.

### 3.2 Photocatalytic activity study of CIP

**Fig. 6:**
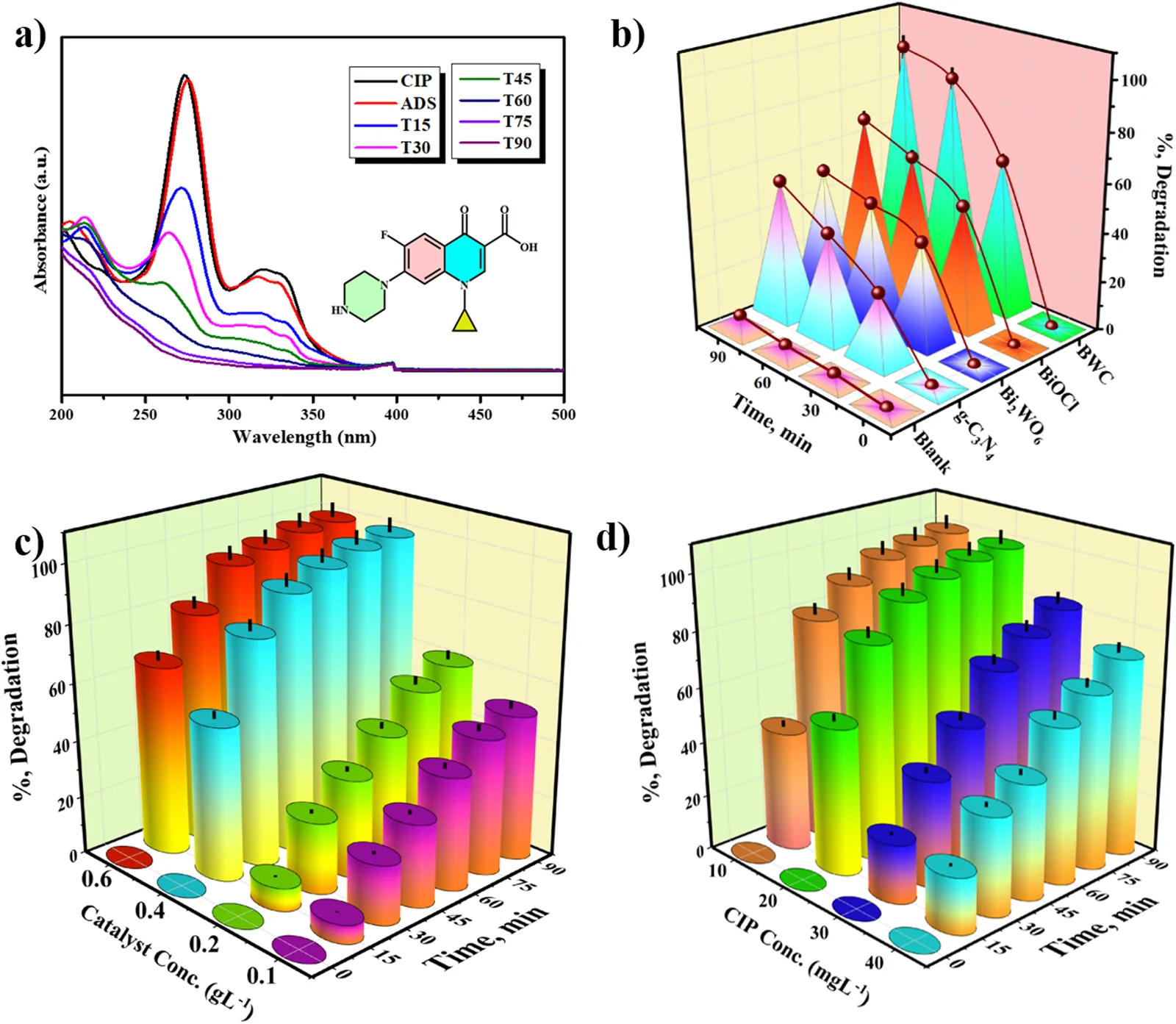
Chemical structure and UV-vis absorption spectrum of CIP (a), effect of catalyst type (b), BWC dosage (c), and initial CIP concentration (d) on the photocatalytic degradation efficiency of CIP. Experimental conditions: CIP concentration = 20 mg L^−1^, catalyst loading = 0.4 g L^−1^, and irradiation time = 0–90 min.

The photocatalytic degradation study of ciprofloxacin (CIP) was systematically investigated by observing the UV-vis spectra. The aqueous solution of CIP showed a pronounced absorption maximum at 274 nm, due to the n → π* and π → π* transitions of its aromatic unit. After adding the photocatalyst in the dark, a minor loss in absorbance (∼6%) was observed, suggesting possible CIP adsorption on the surface of the catalyst. Upon sunlight exposure, the absorption maxima of CIP at 274 nm gradually decreased, leading to complete mineralization after 90 min of irradiation (Fig. 6a).

The photocatalytic study using various catalysts such as BiOCl, Bi_2_WO_6_, g-C_3_N_4,_ and the BWC heterostructure for CIP over 90 min of sunlight exposure is given in Fig. 6b. When CIP alone was exposed to sunlight for 90 min, it showed minimal degradation of ∼6%, confirming that photolysis makes a negligible contribution to CIP degradation. The ternary BWC heterostructure exhibits significant efficiency with almost complete degradation of CIP within 90 min. However, individual catalysts, pristine BiOCl, Bi_2_WO_6_, and g-C_3_N_4,_ exhibit lower efficiencies of 68, 54, and 56%, respectively, compared to the ternary heterostructure, reflecting their poor charge separation abilities. The superior photocatalytic performance of the composite is attributed to the well-developed dual S-scheme heterojunction among BiOCl, Bi_2_WO_6_, and g-C_3_N_4_, which enables efficient charge separation and facilitates the degradation process. These data were also supported by total organic carbon (TOC) measurement. For a 20 ppm CIP solution, the TOC value before the photocatalytic reaction was estimated to be 13.938 ppm (Fig. S3, SI). After the photocatalytic treatment, the TOC was reduced to 2.816 ppm, demonstrating a significant decrease in organic carbon as the CIP molecules, as well as organic intermediates, are degraded into carbon dioxide and water.

#### 3.2.1 Influence of catalyst dosage

To determine the optimum catalyst loading, the photocatalytic degradation of ciprofloxacin was examined at varying BWC dosages from 0.1 to 0.6 g L^−1^ at a constant CIP concentration of 20 mg L^−1^. As presented in Fig. 6c, with an increase in catalyst loading, photocatalytic degradation efficiency was enhanced, referring to the fact that the presence of more surface unsaturation/energy. With a catalyst dosage of 0.4 g L^−1^ and an exposure time of 90 min practically led to almost complete mineralization. The highest degradation of CIP was achieved with a catalyst dosage of 0.6 g L^−1^ and a reaction duration of 75 min; however, considering the minimum atom utilization, the BWC dosage of 0.4 g L^−1^ was chosen as the optimal, as it delivers a practical, sustainable, and more efficient balance between catalytic performance and materials used.

#### 3.2.2 Influence of initial CIP concentration

The relationship between the initial CIP concentration and the photocatalytic degradation activity of BWC is depicted in Fig. 6d. The influence of CIP concentration was investigated between 10–40 mg L^−1^ on the photocatalytic behaviour. Complete degradation was achieved at 10 mg L^−1^ concentration of CIP with an optimized catalyst dosage of 0.4 g L^−1^ within an exposure time of 75 min due to abundant active sites and adequate ROSs. With a CIP concentration of 20 mg L^−1^ almost complete mineralization was achieved within 90 min using an optimized amount of catalyst. However, exceeding the CIP concentration beyond 20 mg L^−1^, *i.e.*, for 30 and 40 mg L^−1^, the degradation efficiency was 83% and 72%, respectively. The observed reduction in degradation efficiency at higher CIP concentrations can be attributed to the excessive number of pollutant molecules present in the reaction medium. Increased CIP concentration limits light penetration through the solution, occupies a larger fraction of the available active sites on the photocatalyst surface, and results in insufficient reactive oxygen species relative to the pollutant load. Consequently, the photocatalytic performance of the BWC heterostructure decreases with increasing CIP concentration. These findings demonstrate that the degradation efficiency is highly dependent on the initial concentration of CIP in the reaction system. At lower concentrations, there is an effective interaction between the substrate and the catalyst, leading to enhanced degradation efficiency. At higher concentrations of CIP, the surface coverage is expected to be higher compared to the accessible active sites under similar reaction conditions, which possibly leads to lower activity compared to lower CIP concentrations.

#### 3.2.3 Evaluation of catalyst heterogeneity and reusability

**Fig. 7:**
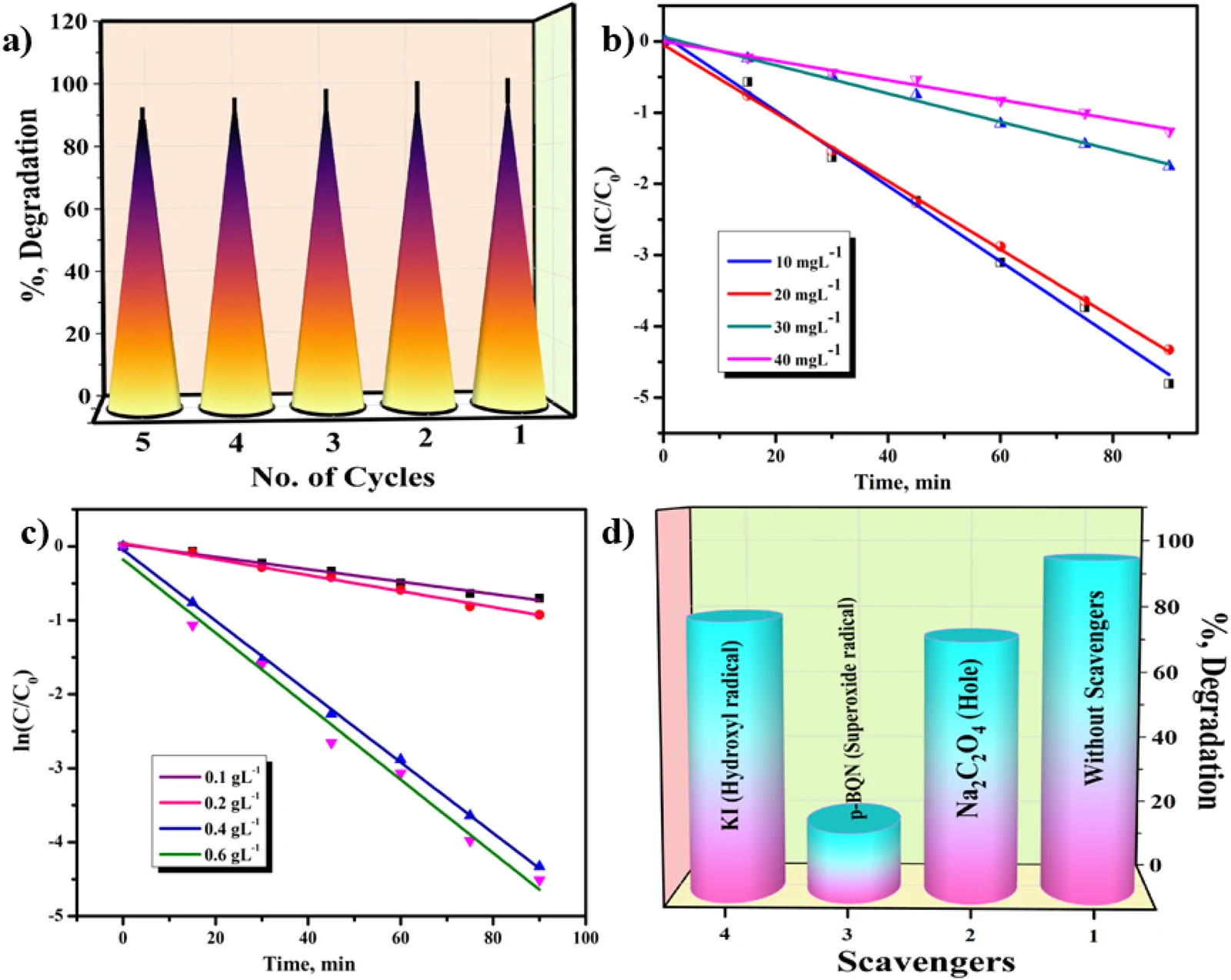
Recyclability performance of the BWC (a), first-order kinetic study (b) and (c), and reactive species trapping experiments using different scavengers (d) for the degradation of CIP; reaction conditions: CIP concentration = 20 mg L^−1^, catalyst loading = 0.4 g L^−1^, and sunlight irradiation time = 0–90 min.

The heterogeneous behaviour of the BWC was monitored by performing the photocatalytic reaction using the solid residue along with the filtrate of the reaction mixture. While using the solid residue, a degradation efficiency of 96% was achieved. The filtrate exhibited 7% degradation value, which is due to the photolysis of CIP molecules with exposure to light, indicating the heterogeneous nature of the CIP degradation process. The stability of the BWC heterostructure was analysed through multiple cycles of experiments. As shown in Fig. 7a, the catalyst showed excellent reusability across five consecutive cycles. In the first cycle, the BWC attained ∼96% mineralization of CIP. The catalyst exhibited a slight reduction upon repeated use, reaching an efficiency of 87% after the fifth cycle. The decline in photocatalytic performance is likely to be associated with surface fouling caused by residual reaction byproducts, along with minor catalyst loss during successive recovery steps. However, the structural integrity of the catalyst remained intact and maintained its activity across all five cycles, proving its robust nature towards multicycle applications.

#### 3.2.4 Kinetic study

A detailed kinetic study of CIP degradation was carried out by varying both the amount of BWC photocatalyst and the initial concentration of CIP. The photocatalytic degradation of CIP exhibited excellent agreement with the first-order kinetic equation, which is given by:

2 Ln *C*_*t*_ = −*kt* + ln *C*_0_

3 Ln(*C*_*t*_/*C*_0_) = −*kt*

here, the initial CIP concentration was denoted by *C*_0_, where *C*_*t*_ presents the concentration at time *t*, *t* is the measured reaction time in min, and *k* is the rate constant.

The graph of ln(*C*_*t*_/*C*_0_) against time is presented in Fig. 7b and c, which yielded straight lines confirming the first-order behaviour of reaction kinetics. The strong linear fitting with *R*^2^ > 0.98 listed in Table 1 further confirms the first-order kinetic model. The apparent rate constants obtained from the slopes of linear plots showed a minor decrease with a rise in initial CIP concentration. The observed decrease in efficiency may be due to saturated surface coverage, restricting the interaction between CIP and BWC heterostructure. The reaction rate constants exhibited a positive correlation with catalyst dosage at a constant CIP concentration. This enhancement arises from the increased active surface area and the availability of more photo-catalytically active sites.

**Table 1:** Variation of the apparent rate constant (*k*) with BWC dosage and initial CIP concentration during the photocatalytic degradation process

Sl. no.	CIP (mg L^−1^)	BWC (g L^−1^)	Rate constant (*k*, min^−1^)	*R* ^2^
1	10	0.4	0.0529	0.995
2	20	0.4	0.0478	0.999
3	30	0.4	0.0198	0.994
4	40	0.4	0.0137	0.992
5	20	0.1	0.0084	0.990
6	20	0.2	0.0108	0.991
7	20	0.6	0.0496	0.990

#### 3.2.5 Scavengers study

The scavenging experiment offers crucial information about the major reactive species responsible for the photocatalytic mineralization. Radical trapping experiments were conducted to evaluate the contribution of different ROS. As shown in Fig. 7d, potassium iodide, sodium oxalate, and *p*-benzoquinone were used as scavengers for hydroxyl (˙OH), photogenerated holes (h^+^), and superoxide radicals (˙O_2_^−^), respectively.^44^ As evident from the degradation results, the addition of *p*-benzoquinone (BQ) caused a pronounced decrease in the degradation efficiency from 96% to 20%. This reduction in degradation efficiency confirms that superoxide radicals (˙O_2_^−^) are the primary reactive species governing the photocatalytic degradation of CIP. In the presence of sodium oxalate, the degradation efficiency decreased to approximately 74%, indicating that photogenerated holes (h^+^) also make a less significant contribution to the degradation process. The introduction of KI to the reaction mixture led to a minor decrease in activity to ∼80%, indicating that hydroxide radicals have a limited contribution in the degradation pathway. Overall, these results confirm that the photocatalytic degradation of CIP is chiefly controlled by a superoxide radical-mediated oxidation process.

#### 3.2.6 Intermediates identification and plausible degradation pathway

**Scheme 3:**
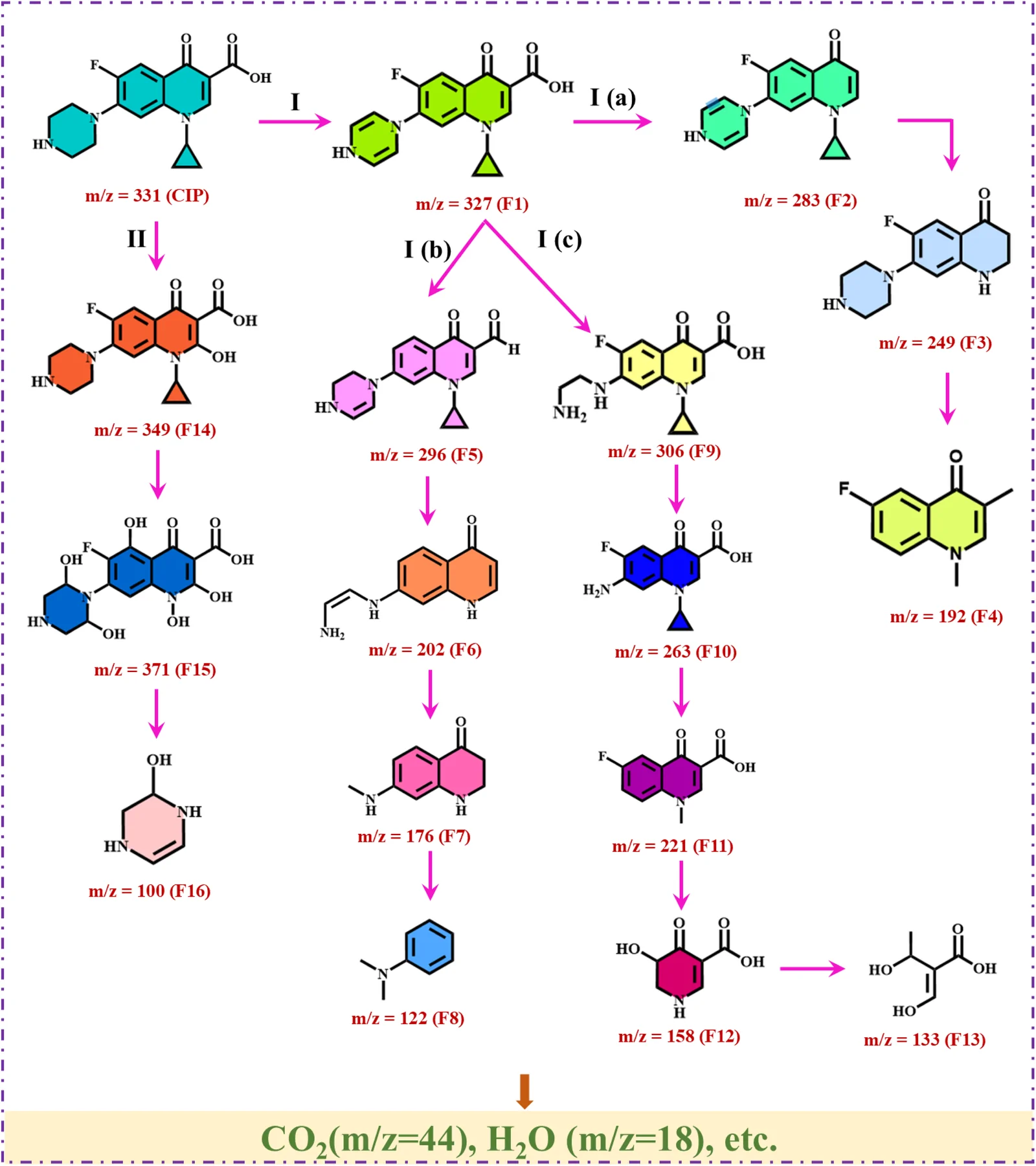
Plausible degradation pathway of CIP degradation.

To elucidate the degradation mechanism, the intermediates produced during CIP photodegradation were analyzed using LC-MS. The detailed mass spectral data corresponding to the detected intermediates are shown in Fig. S4 (SI). CIP comprises various electron-rich functional groups, such as aromatic, keto, carboxyl, fluorine, and piperazinyl groups, which are major sites for the oxidative attack by reactive organic species (ROS) such as photogenerated holes, hydroxyl radicals, and superoxide radicals. Based on the reactive intermediates and their corresponding *m*/*z* values, along with data available in the literature, two pathways (paths I and II) were proposed, as elucidated in Scheme 3.

Path I depicts the oxidation of the piperazinyl group in the CIP molecule (*m*/*z* = 331), forming the intermediate F1 (*m*/*z* = 327). Subsequent decarboxylation of F1 through path I(a) leads to the development of F2 (*m*/*z* = 283). After that, cleavage of the cyclopropyl ring occurs, forming F3 (*m*/*z* = 249), followed by rupture of the piperazinyl ring, yielding fragment F4 (*m*/*z* = 192). Intermediate F1 further undergoes reduction of the carboxylic acid group and the cleavage of the C–F bond through path I(b) to produce fragments such as F5 (*m*/*z* = 296). Subsequent breakdown of the C–C bond of the piperazinyl group and aldehyde group leads to the formation of F6 (*m*/*z* = 202), F7 (*m*/*z* = 176), and F8 (*m*/*z* = 122). F1 undergoes C–C bond cleavage of the piperazinyl group through path I(c) to yield F9 (*m*/*z* = 306) and F10 (*m*/*z* = 263). Then these fragments undergo cleavage of the cyclopropyl ring and attack of the hydroxyl group on the C–F bond, generating low molecular weight species such as F11 (*m*/*z* = 221) and F12 (*m*/*z* = 158). Then the breakdown of the dihydropyridine ring in F12 provides an open structure, F13 (*m*/*z* = 133). Path II involves the attack of the hydroxyl radical on the CIP molecule, providing F14 (*m*/*z* = 349) and F15 (*m*/*z* = 371). Eventually, all fragments undergo successive oxidation, decarboxylation, and bond cleavage, leading to the complete mineralization of CIP, forming CO_2_ (*m*/*z* = 44) and H_2_O (*m*/*z* = 18).

#### 3.2.7 Elucidation of the dual S-scheme photocatalytic mechanism

**Scheme 4:**
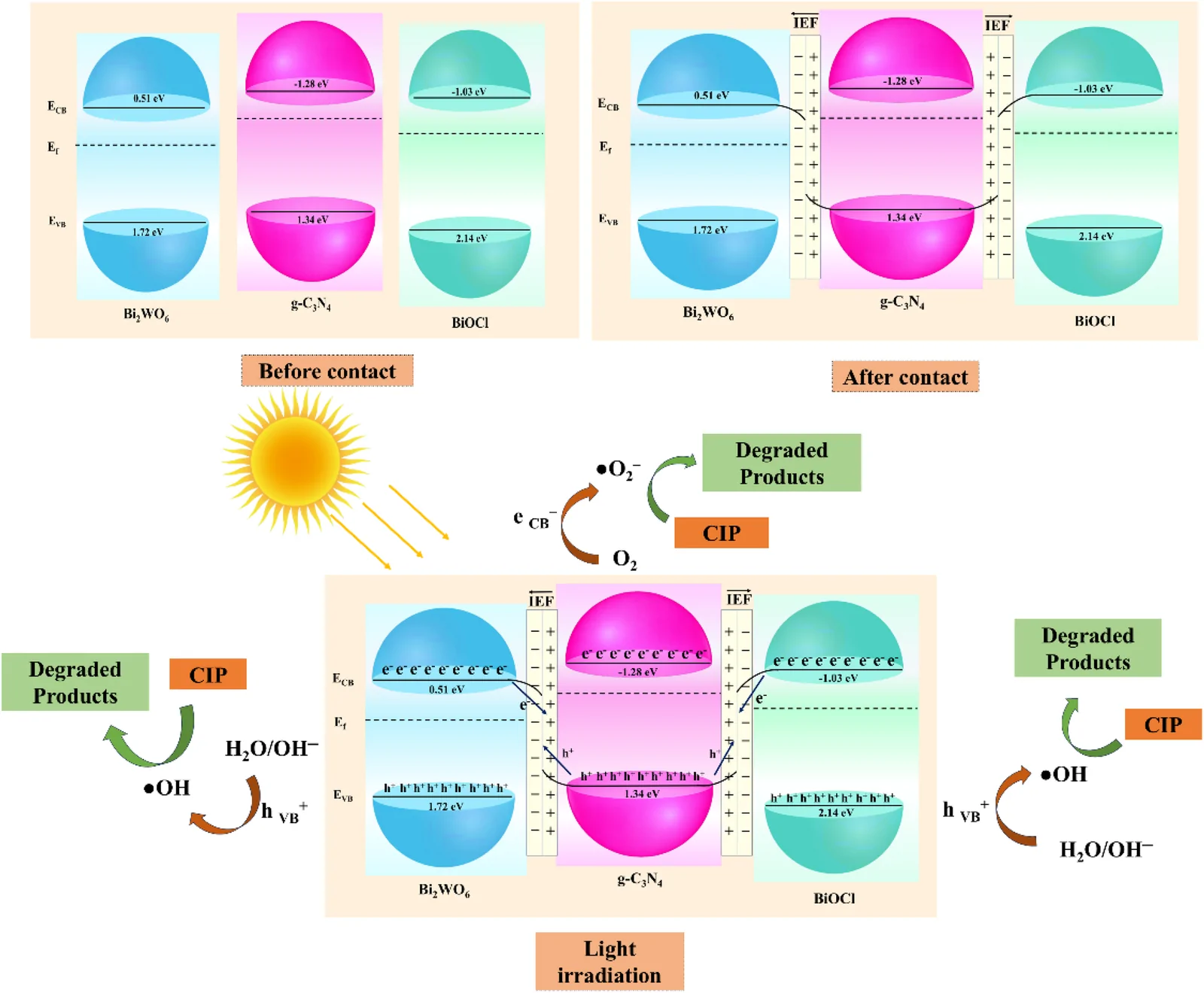
Proposed photocatalytic mechanism and dual S-scheme charge migration pathway for CIP degradation over BWC.

The electronic band structure and band alignment results suggest the formation of a dual S-scheme heterojunction, which governs the charge transfer process within the BiOCl/Bi_2_WO_6_/g-C_3_N_4_ (BWC) heterojunction, as shown in Scheme 4. Prior to the formation of the heterojunction, BiOCl, Bi_2_WO_6_, and g-C_3_N_4_ possess distinct Fermi energy levels and band-edge positions, which provide the driving force for charge redistribution upon interfacial contact.^45^ Upon interfacial contact, electrons migrate from g-C_3_N_4_ to BiOCl and Bi_2_WO_6_ until Fermi level equilibrium is established, leading to interfacial charge redistribution and the generation of an internal electric field across the heterojunction interfaces.^46,47^ After light exposure, photogenerated charge carriers are produced in all the semiconductors. Due to the collective effect of the coulombic force of attraction and strong internal electric field, electrons in the conduction band of Bi_2_WO_6_ and BiOCl recombine with the holes in the valence band of g-C_3_N_4_. Due to this selective recombination, most reactive electrons of the g-C_3_N_4_ and holes of BiOCl and Bi_2_WO_6_ are preserved for the reaction. The electrons retained in the conduction band of g-C_3_N_4_ react with dissolved oxygen to generate superoxide radicals (˙O_2_^−^), whereas the holes accumulated in the valence bands of BiOCl and Bi_2_WO_6_ oxidize surface-adsorbed H_2_O and/or OH^−^ ions to produce hydroxyl radicals (˙OH). These highly reactive oxygen species subsequently attack the CIP molecules, leading to their progressive degradation into intermediates and ultimately mineralized simpler by-products.

## 4. Conclusion

This work presents the photocatalytic mineralization of fluoroquinolone antibiotic CIP molecules using a synthesized ternary BWC heterojunction under natural sunlight exposure. The developed material was broadly characterized for the analysis of structural and morphological properties. Complete mineralization of CIP was observed, and the key reaction parameters were varied for the analysis of their effect on the photocatalytic process. The dual S-scheme BWC heterojunction exhibited superior performance, explaining the synergistic effect of efficient charge separation and suppressed electron–hole recombination. The scavenging experiments confirmed that superoxide radical is the major reactive species responsible for the effective decomposition of CIP. The developed heterojunction exhibited good thermal stability along with robust structural durability. Furthermore, the reusability experiments confirmed the negligible loss in activity of the heterojunction catalyst. Overall, the present study establishes heterojunction engineering as an effective strategy to overcome the fundamental shortcomings associated with conventional photocatalysts. The BWC photocatalyst exhibits superior photocatalytic performance, enhanced stability, and strong responsiveness under solar irradiation, demonstrating its suitability for sustainable water treatment applications. These results emphasize its potential as an efficient material for the removal of CIP and highlight its applicability in next-generation environmental remediation technologies.

## Author contributions

Laxmipriya Pradhan: material preparation, investigation, methodology, software, writing-original draft, data curation. Mano Ranjan Barik: methodology, conceptualization, writing-review and editing, formal analysis. Sushanta Kumar Badamali: supervision, conceptualization, methodology, writing-review and editing, formal analysis.

## Conflicts of interest

There are no conflicts to declare.

## Data Availability

The data supporting this article have been included as part of the supplementary information (SI). Supplementary information: EDAX pattern of BWC heterojunction (Fig. S1), photoluminescence spectra of BiOCl, Bi_2_WO_6_, g-C_3_N_4_, and BWC heterojunction (Fig. S2), TOC analysis before and after photocatalytic reactions (Fig. S3), LCMS spectra of CIP degradation (Fig. S4), comparative table for the photocatalytic degradation of CIP (Table TS1). See DOI: https://doi.org/10.1039/d6ra06114a.
